# Artificial Heme Enzymes for the Construction of Gold-Based Biomaterials

**DOI:** 10.3390/ijms19102896

**Published:** 2018-09-24

**Authors:** Gerardo Zambrano, Emmanuel Ruggiero, Anna Malafronte, Marco Chino, Ornella Maglio, Vincenzo Pavone, Flavia Nastri, Angela Lombardi

**Affiliations:** 1 Department of Chemical Sciences, University of Napoli “Federico II” Via Cintia, 80126 Napoli, Italy; gerardo.zambrano@unina.it (G.Z.); emmanuel.ruggiero@basf.com (E.R.); anna.malafronte@unina.it (A.M.); marco.chino@unina.it (M.C.); ornella.maglio@unina.it (O.M.); vincenzo.pavone@unina.it (V.P.); 2 Istituto di Biostrutture e Bioimmagini, CNR, Via Mezzocannone 16, 80134 Napoli, Italy

**Keywords:** gold nanoparticles, artificial enzymes, immobilization strategies, catalytic activity, peroxidases, functional biomaterials

## Abstract

Many efforts are continuously devoted to the construction of hybrid biomaterials for specific applications, by immobilizing enzymes on different types of surfaces and/or nanomaterials. In addition, advances in computational, molecular and structural biology have led to a variety of strategies for designing and engineering artificial enzymes with defined catalytic properties. Here, we report the conjugation of an artificial heme enzyme (MIMO) with lipoic acid (LA) as a building block for the development of gold-based biomaterials. We show that the artificial MIMO@LA can be successfully conjugated to gold nanoparticles or immobilized onto gold electrode surfaces, displaying quasi-reversible redox properties and peroxidase activity. The results of this work open interesting perspectives toward the development of new totally-synthetic catalytic biomaterials for application in biotechnology and biomedicine, expanding the range of the biomolecular component aside from traditional native enzymes.

## 1. Introduction

Extensive progress in protein design and engineering enables the construction of artificial metalloenzymes showing efficiency and substrate diversity beyond those of natural systems [1,2,3,4,5,6,7,8,9,10,11,12]. Heme-containing enzymes active in oxidation chemistry are a fascinating source of inspiration for researchers [13]. Indeed, recent outstanding results demonstrated that artificial biocatalysts with new reactivity can be successfully developed, by redesigning, modifying and/or mimicking heme-enzymes. Redesign of heme-proteins, through engineering and/or reconstitution with different porphyrin-like cofactors, allows shaping new functions into native proteins [14,15,16,17,18]. By mimicking Nature’s strategy, directed evolution successfully allows repurposing enzymes, such as Cytochrome P450, thus developing catalysts for reactions not familiar to the biological systems [19,20,21,22,23]. Further, rational design and de novo design approaches have allowed the construction of artificial peroxidases with enzymatic rate constants outperforming those of their natural counterparts [24,25].

The potential of artificial metalloenzymes will be fully exploited once the results from basic science are transferred into specific technological applications, such as in biotechnology and industrial catalysis. Merging the advantages of designed enzymes and immobilization strategies could be an important step toward these goals [26]. In this view, the tools available today for the chemical modification of enzymes have strongly increased the variety of functional biomaterials that can be developed. Nowadays, enzymes are widely used in several biotechnological and biomedical applications, thanks to a variety of strategies implemented for their conjugation and/or immobilization onto different matrices [27]. Conjugation of enzymes to solid supports and/or nanomaterials has been used for the development of bio-devices with a variety of functions, ranging from biosensing, to drug delivery, targeting and imaging [28,29,30]. The utility and effectiveness of these bio-conjugates relies on the combination of the unique properties of the solid supports and/or nanomaterials with those of the biomolecular components [31,32,33,34].

In particular, gold-based materials are well suited for the immobilization of redox-active enzymes [31]. They allow electron exchanges with the biomolecular components thus being of great interest in the field of electrochemical biosensors. Further, gold nanoparticles (AuNPs) have emerged as useful nanomaterials for enzyme immobilization because of their surface chemistry and unique electronic, magnetic and optical properties [35]. In addition, immobilization of enzymes on AuNPs represents a middle ground between heterogeneous (immobilized enzymes) and homogeneous (soluble free enzymes) catalysis [36].

Heme-proteins have been successfully immobilized onto different supports for diverse applications [37,38]. They can rapidly exchange electrons with electrodes, by a direct reversible electron transfer, and Cytochrome *c* is one of the most studied heme-protein in this respect [37]. Further, the immobilization of electro-active enzymes, such as Cytochrome P450, on gold electrodes is a useful strategy for the development of biosensors and screening platforms for drug development [38,39]. Peroxidases and catalases have often been conjugated to AuNPs and to other super-paramagnetic nanoparticles, and the applications of these bio-nano-conjugates have been successfully exploited in immunoassays or biosensor development [33,40]. For example, horseradish peroxidase (HRP) entrapped onto layers of gold nanoparticle-thionine-chitosan absorbed on a glassy carbon electrode (GCE) was applied as signal amplification system, for the electrochemical biosensing of tetrahydrocannabinol [41]. Similarly, by encapsulation on nanogold hollow spheres, HRP was used for signal amplification in the electrochemical detection of hepatitis C virus [42].

Substituting natural enzymes with properly designed metalloenzymes would be valuable for the development of new hybrid and functional biomaterials. First, it would expand the set of available biomolecular components beyond the natural system. Further, fine-tuning the enzyme performances by design should open the way to the construction of nanoparticles/artificial enzyme conjugates tailored for specific applications.

**Figure 1:**
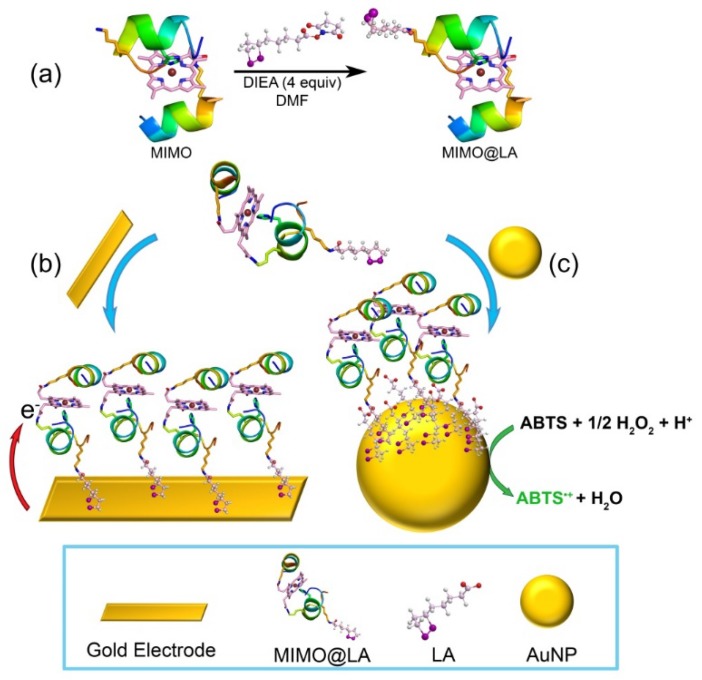
MIMO lipoamide (MIMO@LA): A versatile building block for the construction of synthetic gold-based biomaterials. (**a**) Strategy for MIMO@LA synthesis (DIEA: diisopropylethylamine; DMF: *N*,*N*-dimethylformamide; experimental conditions are reported in Section 3.1). (**b**) Immobilization of MIMO@LA on gold electrode affords a redox active interface. (**c**) Conjugation to gold nanoparticles (AuNPs) gives a catalytic system for substrate oxidation. The oxidation of the chromogenic substrate 2,2′-azino-bis (3-ethylbenzothiazoline-6-sulphonic acid) (ABTS) to ABTS radical cation (ABTS^●+^) is reported as an example.

In this view, we exploited the applications of mimochromes, artificial peroxidases developed by us through a miniaturization approach [1,13,24,43,44,45,46,47,48,49,50,51,52,53,54], for the construction of totally synthetic functional bioconjugates. Their simple structure (Figures 1 and S1), made up by two helical peptide chains sandwiching an iron-porphyrin, allowed shaping a proximal and distal face around the metal cofactor, thus resembling natural heme-proteins [24,50,52]. Starting from the prototype Mimochrome I [43,44,45], in which the presence of two histidine (His) residues in each peptide chains afforded a symmetric *bis*-His hexa-coordinated complex, several rounds of redesign allowed the development of catalytically active derivatives [24,50,54]. In particular, replacement of the axial-coordinating His in one chain, with a smaller residue unable to coordinate the metal ion, provided five-coordinated complexes with an empty site available for substrate binding and transformation [24,50,54]. Several analogs of the mimochrome family have been fully characterized, displaying both reversible Fe(III)/Fe(II) redox behaviors and peroxidase catalytic activity [48,50,52,54]. The properties of Fe(III)-Mimochrome VI (the first developed five-coordinated complex), herein referred to as MC6 [50,52], have already been investigated upon immobilization on electrode surface, by exploiting physical adsorption on either gold electrode coated with decane-1-thiol or on mesoporous conductive films of tin-doped indium oxide (ITO) [52,53].

In this work, a derivative of the mimochrome family, specifically Fe(III)-S6G(*D*)-MC6 (named as MIMO throughout the text), was immobilized on gold-based materials. A crucial issue in the immobilization of enzymes onto different supports is to find the optimal conditions to preserve catalyst functionality [55,56,57]. The covalent attachment of heme-enzymes, through thiol-based self-assembled monolayers (SAMs) on gold, is one of the most applied methods for ensuring enzyme direct electrical contact with electrodes [37,58,59]. For AuNPs, numerous studies have been devoted in finding the optimal solution for interfacing catalytic and redox active enzymes on nanoparticles. In particular, the use of AuNPs in aqueous solutions requires their stabilization in a finely dispersed state [60]. For this purpose, the use of a proper coating moiety is mandatory. Based on these considerations, MIMO was functionalized with lipoic acid (LA) to construct a building block that can easily be grafted onto different gold-based supports, such as gold electrodes or AuNPs (see Figure 1).

Herein, we demonstrate that the artificial catalyst displays quasi-reversible redox properties and peroxidase activity, even upon immobilization. Collectively, the straightforward synthesis of MIMO@LA and the simplicity of the immobilization strategy on gold-based supports offer wide perspectives in biosensing and catalysis.

## 2. Results and Discussion

### 2.1. Synthesis of MIMO@LA

In this work, we explored the feasibility of using LA-modified MIMO for its direct immobilization on gold-containing materials. Amino groups of proteins can be converted into thiols by the use of bi-functional linkers, containing both a thiol and a carboxylic group. Among sulfur-containing bi-functional linkers, LA has been widely applied for the preparation of a variety of stable conjugates, useful for different applications [61,62,63,64,65,66].

MIMO (see Figure S1 for details) was synthesized following a previously developed procedure [50,54]. It was purified to homogeneity by reverse phase—high performance liquid chromatography (RP-HPLC), and electrospray ionization-mass spectrometry (ESI-MS) confirmed the expected molecular weight (3521.2 amu). For a more detailed description of synthetic protocols and results, please refer to Supplementary Materials (Section S2.1 and Figure S2). The UV/Vis spectrum at neutral pH (Figure S3) is representative of a high spin ferric porphyrin, with His-H_2_O axial coordination [50]. Conjugation of MIMO to LA was accomplished through several simple steps (see Scheme S1). The presence of a single Lys residue, in one of the two peptide chains, exposed to the solvent, suggested the use of its side chain as reactive group for coupling to the carboxyl group of LA. To this aim, LA was activated as *N*-hydroxysuccinimide (NHS) ester, and subsequently it was conjugated to MIMO via amide bond formation. The reaction product was purified by RP-HPLC (Figure S4), thus yielding MIMO@LA (yield 34%).

### 2.2. Electrochemical Characterization

**Figure 2:**
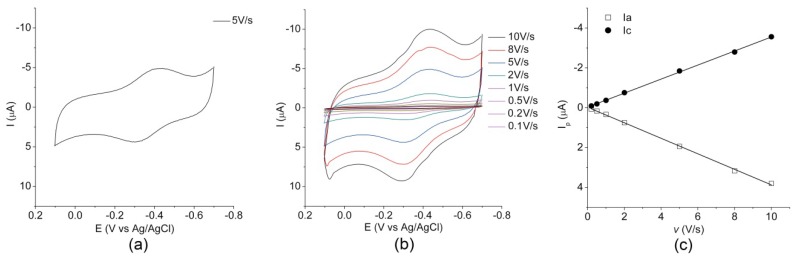
Electrochemical characterization of MIMO@LA. (**a**) Cyclic voltammograms for MIMO@LA on the gold electrode in 10.0 mM phosphate buffer solution, pH 7.0, at scan rate (*ν*) 5.0 V/s. Measurements are reported vs. Ag/AgCl electrode. (**b**) Cyclic voltammograms for MIMO@LA on the gold electrode in 10.0 mM phosphate buffer solution, pH 7.0, recorded in the scan range 0.1–10.0 V/s. (**c**) Peak current intensity dependence (I_p_) versus *v.* I_c_ and I_a_ are the peak current intensities of the cathodic and anodic peaks, respectively.

To verify whether MIMO retains its redox properties upon immobilization on the gold electrode, a comparison of the electrochemical properties of both the immobilized and freely diffusing enzyme was carried out using cyclic voltammetry (CV). The cyclic voltammogram of MIMO@LA immobilized onto the gold electrode, measured at 5.0 V/s scan rate, is reported in Figure 2a. The voltammogram shows two well-defined peaks in the potential region between −700 and 100 mV (vs. Ag/AgCl). The cathodic peak (*E*_pc_) and the corresponding anodic peak on the reverse scan (*E*_pa_) are located at −391 mV and −313 mV (vs. Ag/AgCl), respectively. For a detailed electrochemical analysis, CV scans were performed at various *v*, ranging from 0.1 to 10.0 V/s (Figure 2b). The linear increase of the cathodic and anodic peak current (I_p_) with increasing scan rate (Figure 2c), confirmed the presence of a diffusion-less redox-active enzyme immobilized onto the electrode surface [37]. According to the literature, from the slope of the cathodic peak currents versus the scan rate it is possible to estimate the enzyme coverage of the functionalized electrode surface [67]. The coverage Γ (mol·cm^−2^) of the functionalized electrode surface was estimated 20.4 pmol·cm^−2^. This value is similar to the value of 23.5 pmol·cm^−2^ obtained for the parent mimochrome adsorbed through hydrophobic interactions on a gold electrode coated with decane-1-thiol [52]. The peak potential separation (ΔE_p_) of about 78 mV indicates that the immobilized MIMO@LA undergoes a quasi-reversible electrochemical reaction [68]. The formal reduction potential *E*°′ of the Fe(III)/Fe(II) couple, derived from the half-wave potential *E*_1/2_ = (*E*_pc_ + *E*_pa_)/2, was −143 ± 5 mV vs. SHE and −352 mV vs. Ag/AgCl.

The cyclic voltammogram of the freely diffusing molecule measured at a scan rate of 5.0 V/s (Figure S5) shows two well-defined current peaks. The cathodic and anodic peaks (*E*_pc_ = −352 mV and *E*_pa_ = −300 mV vs. Ag/AgCl, respectively) are similar in shape and magnitude, with a current intensity ratio (*i*_pa_/*i*_pc_) close to unity, and a ΔE_p_ in line with the expected theoretical value for a reversible mono-electronic process [68]. The formal reduction potential *E*°′ was −117 ± 3 mV (vs. SHE and −326 mV vs. Ag/AgCl. This value is in good agreement with those previously reported for MC6 and other freely diffusing mimochrome molecules [54,58].

The *E*°′ value of the immobilized enzyme was found to be slightly more negative than for the freely diffusing enzyme. The observed small shift could be related to small changes in the environment occurring when the enzyme is covalently anchored on the surface. All these results demonstrated that MIMO retains its ability to exchange electrons with an electrode surface upon immobilization. Interestingly, the CVs remained essentially unchanged on consecutive potential cycling, thus indicating a good stability of the enzyme-modified electrode. 

### 2.3. MIMO@LA Catalytic Activity on AuNPs

#### 2.3.1. Synthesis and Characterization of MIMO@LA@AuNPs 

AuNPs were prepared in situ [69] by reduction of tetrachloroauric acid (HAuCl_4_) in the presence of trisodium citrate, according to the Turkevich method [70]. The citrate-stabilized AuNPs were subsequently functionalized with MIMO@LA by ligand exchange reaction with the lipoic acid moiety. To control the density of the biomolecule on the AuNPs surface and reduce steric hindrance, a mixture of MIMO@LA and LA in dimethylsulfoxide (DMSO) (with a 6:1 LA:MIMO@LA ratio) was added to AuNPs-citrate in water (pH 11). After incubation, the reaction mixture was centrifuged to remove excess of MIMO@LA and LA; the supernatant was decanted; and the pellet was resuspended in NaOH solution (pH 11). This procedure of centrifugation/redispersion step was repeated several times. Complete removal of free MIMO@LA enzyme was ascertained by the absence of any catalytic activity in the supernatant (see Section 2.3.2). This procedure allowed obtaining the purified conjugate MIMO@LA@AuNPs, which was finally resuspended in NaOH solution (pH 11) and promptly used for subsequent experiments.

**Figure 3:**
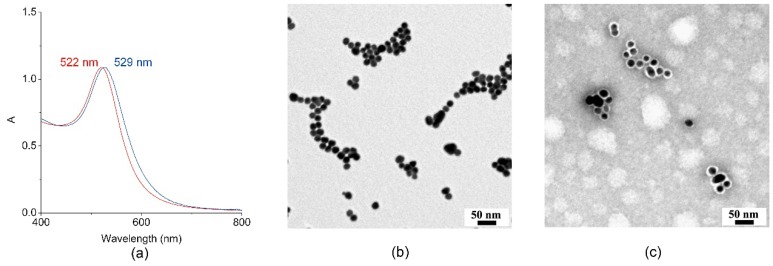
Characterization of MIMO@LA@AuNPs. (**a**) Visible spectra of citrate-capped AuNPs (red line) and of the bioconjugate MIMO@LA@AuNPs (blue line). (**b**) Transmission Electron Microscopy (TEM) image of citrate-capped AuNPs. (**c**) TEM image of the bioconjugate MIMO@LA@AuNPs. The staining with 1% uranyl acetate allowed highlighting the protein shell as a white halo around each AuNP.

Conjugation of MIMO@LA to AuNPs was ascertained by Visible spectroscopy. Figure 3a reports the superposition of the visible spectra of citrate-stabilized AuNPs (red line) and of the MIMO@LA@AuNPs bioconjugate (blue line). Citrate-stabilized AuNPs showed the characteristic Surface Plasmon Resonance (SPR) band with a maximum at 522 nm [71]. As expected, conjugation of the artificial enzyme MIMO@LA to AuNPs caused a red-shift of the SPR band from 522 to 529 nm. The SPR band is sensitive to refractive index changes in the proximity of the nanoparticle surface [71]. The observed red-shift, and no significant SPR band broadening is indicative that, upon MIMO@LA coating, AuNPs remained well dispersed without any aggregation in the colloidal suspension.

Figure 3 reports a comparison of the Transmission Electron Microscopy (TEM) images of citrate-stabilized AuNPs (Figure 3b) and MIMO@LA@AuNPs (Figure 3c), respectively. The citrate-stabilized AuNPs appeared uniform in size and shape, with an average diameter of 16 ± 0.5 nm, as assessed by the size distribution histogram (Figure S6a). TEM image of MIMO@LA@AuNPs, stained by 1% uranyl acetate [72] (Figure 3c), showed that, upon enzyme conjugation, the size of the AuNPs gold core was retained upon modification with MIMO@LA (Figure S6b). The presence of segregated patches of nanoparticle in the TEM images is due to the deposition process [73].

An average diameter of 28 ± 0.5 nm was measured for the MIMO@LA@AuNPs conjugate (Figure S6c), with a protein shell thickness of 5.0 ± 0.5 nm.

To quantify the amount of MIMO@LA loaded on each AuNPs, inductively coupled plasma mass spectrometry (ICP-MS) was used. The concentration of MIMO in the MIMO@LA@AuNPs solution was determined by quantifying the heme moiety on the basis of the total iron content of the sample (10 µM). Given an AuNPs concentration of 8 nM (see Materials and Methods), an average loading of ≅1200 MIMO molecules per nanoparticle was obtained. 

The maximum theoretical number of protein molecules (*N*_max_) that can be loaded on each spherical AuNP was calculated through geometrical considerations, as previously reported by Mattoussi et al. [74], using the following equation:

(1) $$N_{max}=0.65\times \frac{\left(R_{complex}^{3}-R_{AuNP}^{3}\right)}{R_{protein}^{3}}$$

where R_complex_ is the AuNP-protein complex radius, R_AuNP_ is the gold nanoparticle radius, and R_protein_ is the radius of the protein. This equation takes into account the filling factor for hard sphere, calculated to be 0.65 [75]. R_AuNP_ comprises the sum of the AuNP radius (8 nm, as obtained from TEM data) and the thickness of the layer composed by the side chain of Lys residue and LA (estimated to be around 2 nm, by considering both chains in an all-*trans* extended conformation, see Figure S7). R_complex_ comprises the sum of R_AuNP_ and the diameter of the MIMO protein. Looking at the model structure (see Figure S7), MIMO molecule has a cylindrical shape, with dimensions of 1.6 nm in diameter and 2.6 nm in length. To take into account all possible randomly oriented cylinder molecules with respect to the AuNP, the radius of gyration (*R_G_*) [76] for MIMO was calculated (see Materials and Methods), and the value of 2 × *R*_G_ (1.8 nm) was used for MIMO diameter in the calculation of R_complex_. This calculation afforded an estimated theoretical number (*N*_max_) of ≅ 570 MIMO per nanoparticle. This value is almost half of that obtained from experiments ICP-MS analysis (MIMO:AuNP ≅ 1200). Furthermore, the calculated MIMO diameter (1.8 nm) is almost half of protein shell thickness measured by TEM. Thus, these analyses suggest the formation of a MIMO double layer around each AuNP, likely due to protein–protein interactions.

#### 2.3.2. Catalytic Activity of MIMO@LA@AuNPs 

To verify whether MIMO acts as a competent catalyst even upon immobilization onto AuNPs, the peroxidase activity of the conjugate was assayed, using 2,2′-azino-bis (3-ethylbenzothiazoline-6-sulphonic acid) (ABTS) as substrate and H_2_O_2_ as oxidizing agent, under the optimal experimental conditions (50 mM phosphate buffer pH 6.5, 50% 2,2,2-trifluoroethanol (TFE) *v*/*v*) previously found for mimochromes [24,50,54]. 

It should be noticed that both stability of the AuNPs colloidal dispersion and catalytic performances strongly depend on pH. Thus, the optimal pH for colloidal stability could be sometimes far from the optimal pH range for catalysis [77]. In the case of MIMO@LA@AuNPs conjugate, colloidal stability is retained when the conjugate is placed in the environmental condition optimized for enzyme catalysis. In fact, no significant changes were observed in the visible spectra of the colloidal suspension, apart from a small blue-shift (2 nm) of the SPR band absorbance maximum, probably due to the presence of TFE (Figure S8).

**Figure 4:**
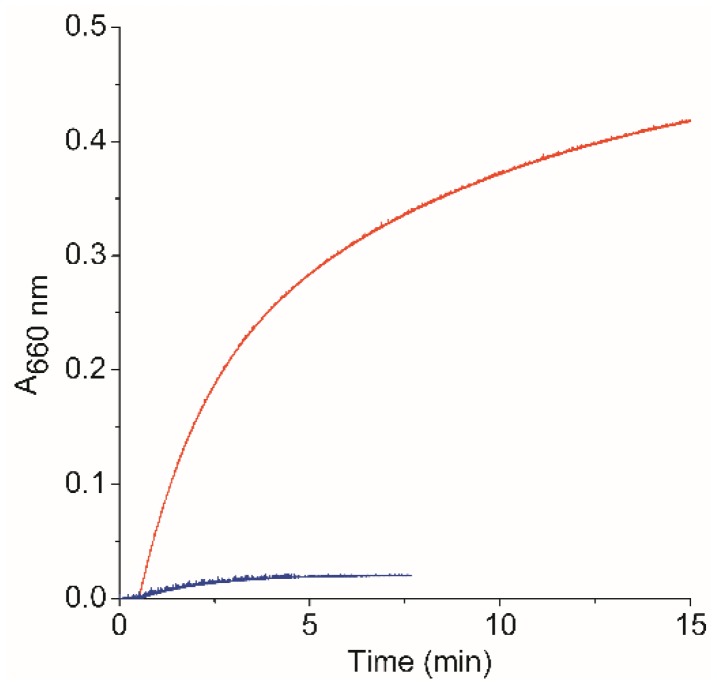
Catalytic activity of MIMO@LA@AuNPs. Time-dependent absorption at 660 nm of ABTS^+●^ during ABTS oxidation, as observed in the resuspended pellet (red curve) and the supernatant (blue curve) of the MIMO@LA@AuNPs solution, after centrifugation. Reaction conditions were: 5.0 mM ABTS and 10.0 mM H_2_O_2_ in 50.0 mM phosphate buffer pH 6.5, 50% 2,2,2-trifluoroethanol (TFE) (*v*/*v*).

Catalytic assays were first aimed at verifying that the activity of the conjugate was only related to the AuNPs immobilized form of the artificial MIMO enzyme. To this aim, a kinetic control assay was done after various centrifugation cycles, on both the supernatant and on the resuspended pellet. Figure 4 shows the progress curve for the oxidation of ABTS, catalyzed by the AuNP-immobilized enzyme, by following the absorbance of the oxidation product ABTS radical cation (ABTS^+●^) at 660 nm as a function of time. Significant activity was detected only in the nanoparticles resuspended pellet, containing MIMO@LA@AuNPs. In the supernatant, activity is near to background autoxidation levels. This demonstrated that the catalyst was immobilized onto nanoparticles, and confirmed the absence of free MIMO@LA enzyme in solution.

**Figure 5:**
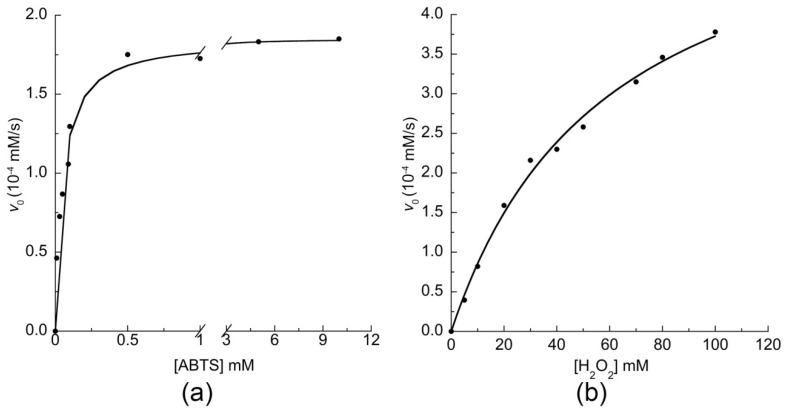
Peroxidase activity of MIMO@LA@AuNPs: (**a**) initial rate dependence towards ABTS concentration; and (**b**) initial rate dependence towards H_2_O_2_ concentration. Reaction conditions were: 50.0 mM phosphate buffer pH 6.5 50% TFE (*v*/*v*); MIMO@LA@AuNPs enzyme concentration 3.5 × 10^−7^ M. For the experiments performed at variable ABTS (**a**), the H_2_O_2_ concentration was 30.0 mM. For the experiments performed at variable H_2_O_2_ (**b**), the ABTS concentration was fixed at 5.0 mM.

This result is a first evidence that the artificial MIMO enzyme is still active when conjugated to the AuNPs. To obtain a detailed kinetic analysis of the MIMO@LA@AuNPs conjugate, the kinetic parameters of the enzyme were determined by varying H_2_O_2_ concentration using fixed concentrations of ABTS, and vice versa, in the same conditions used for the previous experiment. The initial rates of ABTS oxidation (*v*_0_) were plotted as a function of both substrate concentrations (Figure 5). Interestingly, the enzyme activity followed a typical Michaelis–Menten kinetics, as observed for the freely diffusing enzyme (Figure S9).

Table 1 reports the *k*_cat_ (turnover frequency) and *K*_M_ (Michaelis–Menten constant) values for the ABTS oxidation, determined for both the immobilized and free enzyme. In the freely diffusing form, MIMO shows a catalytic activity comparable to that of the previous analog MC6 [50]. A decrease in the *k*_cat_ value together with a slight increase in the *K*_M_ values was instead observed when comparing the suspension of MIMO@LA@AuNPs to the freely diffusing enzyme.

This result, although unfavorable, is not surprising. In fact, the catalytic efficiency of natural enzymes can broadly change upon immobilization onto different surfaces and/or AuNPs [36,78,79].

For example, the effect of the immobilization and/or conjugation to nanoparticles in enhancing or reducing the catalytic activity, relatively to the freely diffusing enzyme, has been deeply analyzed for horseradish peroxidase (HRP) [80,81,82]. In particular, the effect of the hydrodynamic diameter and nanoparticle curvature on the reactivity of HRP when conjugated to AuNPs has recently been reported [82]. Similar to our system, HRP showed reduced catalytic activity toward ABTS oxidation, and this finding was demonstrated to strongly correlate with the size of AuNPs. Indeed, the *k*_cat_ value decreases when moving from the free enzyme (800 s^−1^) to the AuNP-HRP bioconjugate as a function of AuNPs diameter (AuNP-HRP diameter 25 nm; *k*_cat_: 500 s^−1^; AuNP-HRP diameter 40 nm, *k*_cat_: 200 s^−1^). Moreover, the *K*_M_ value for ABTS doubled upon HRP conjugation (free HRP, *K*_M_ ≅ 3 mM_;_ AuNP-HRP diameter 18 nm, *K*_M_ ≅ 5.5 mM) [82]. Diffusion–collision theory coupled with experimental studies allowed to hypothesizing that two main factors affect the catalytic parameters of these AuNP-HRP bioconjugates: (1) influence of the bioconjugate sizes on the diffusion kinetics, which impacts the *k*_cat_ values; and (2) changes in the secondary structures of the HRP active site, depending on the AuNP curvature, which affects *K*_M_ values.

Changes in the kinetic performances of enzymes upon immobilization onto AuNPs have been also related to enzyme multilayer formation [83]. Even though enzyme accumulation and crowding onto AuNPs could have favorable effects on enzyme kinetics, due to the stabilization of both the enzyme active conformation and colloidal suspension, they can also have negative effects [83]. When enzyme multilayer formation occurs, the activity of enzymes in the inner layers can be affected by the poor accessibility of substrates, by the competition with substrates of the outer layer enzymes and/or by inhibition of enzyme conformational changes during catalysis for steric hindrance [83].

According to these previously reported results, we hypothesize that the observed reduction of MIMO catalytic efficiency upon immobilization onto AuNPs is related to the MIMO double layer formation around each AuNP (see previous section). This hypothesis is supported by preliminary results obtained on a different MIMO@LA@AuNPs conjugate preparation, in which the size of AuNPs was decreased (13 nm diameter, data not shown). In this preparation, to reduce MIMO multilayer formation, the synthetic procedure included, as final step in the conjugation procedure, a prolonged washing step with TFE on the MIMO@LA@AuNPs conjugate. This washing procedure was performed because, thanks to the high solvation properties of TFE and solubility of MIMO in this solvent, it could inhibit MIMO-MIMO interactions and/or aggregation on the AuNP surface, thus disfavoring multilayer formation. Indeed, in this preparation, conjugation of MIMO@LA to AuNP was found to afford an average loading of ≅470 MIMO molecules per nanoparticle, close to the maximum theoretical number of protein molecules expected for a monolayer formation (*N*_max_ = 570, see above).

Remarkably, this conjugate displayed higher kinetic parameters than those for the multilayer preparation, with an almost ten-fold increase of the *k*_cat_ value (*k*_cat_ = 15.2 ± 2 s^−1^; *K*_M_ = 25.5 ± 6 × 10^−5^ M; k_cat_/*K*_M_ = 59.6 ± 20 mM^−1^ s^−1^), and only 30-fold decrease from the freely diffusing MIMO. Assuming that, in the double-layer MIMO@LA@AuNPs conjugate, only the outermost enzyme layer is the active one, the measured *k*_cat_ value could only double (*k*_cat_ ≅ 3.4 s^−1^). Even with this assumption, *k*_cat_ does not reach the value obtained for the monolayer preparation, thus suggesting that multilayer formation negatively influenced the catalyst performances. We cannot exclude that other factors such as influence of the bioconjugate sizes on the diffusion kinetics, limited diffusion of the substrates to the active site, partial loss of the catalyst active structure, and length of the linker could influence the variations in the catalytic parameters of MIMO when conjugated to the AuNPs. A deep study on the factors affecting the catalytic parameters of the MIMO enzyme when conjugated to AuNPs is underway.

Interestingly, the overall results strongly support the feasibility of using mimochrome artificial peroxidases for the development of AuNP-based biomaterials. It should be highlighted that the presence of the MIMO@LA coating layer positively impacts the colloidal stability, even at neutral/slight acidic pH conditions. This represents a key point in the preparation and use of enzyme-AuNP conjugates to avoid AuNP coalescence while preserving enzyme activity.

## 3. Materials and Methods

All solvents, used in the synthesis and purification, were anhydrous and HPLC grade, respectively, and were supplied by Romil (Cambridge, UK). All buffer solutions were made by using water with a HPLC purity grade (Romil); phosphate salts (monobasic and dibasic) for buffers preparation, NHS, THF (tetrahydrofuran), uranyl acetate dihydrate and H_2_O_2_ (30%, *v*/*v*) were provided by Fluka; TFE was supplied from Romil. The DCC (*N,N*′-Dicyclohexylcarbodiimide) as well as HAuCl_4_ solution (30% *w*/*w*) were obtained from Sigma Aldrich (Taufkirchen, Germany). The electrodes, the electrochemical cell, the alumina and the diamond powder for cleaning the electrodes are from BASi (West Lafayette, IN, USA).

Iron content of MIMO@LA@AuNPs solution was quantified by ICP-MS analysis, using Aurora Bruker M90 instrumentation (Bremen, Germany). Aqua regia digestion procedure (90 °C, overnight) was adopted for organic component and gold degradation. Iron concentration was estimated at 0.55 mg/L (~9.85 × 10^−6^ M). UV-vis analysis was performed on Cary Varian 50 Probe UV Spectrophotometer (Varian, Palo Alto, CA, USA). In all analyses, 1 cm path length quartz cuvettes were used. All the data were analyzed by using the Origin Pro 8 (Origin Lab Corporation, Northampton, MA, USA) and the Kaleidagraph software (version 4.1.1, Synergy Software, Reading, PA, USA).

### 3.1. Synthesis of MIMO@LA

MIMO was modified at the side chain of Lys11 with lipoic acid to allow its subsequent conjugation to AuNPs. To this aim, the carboxyl group of LA was first activated as NHS ester, by using dicyclohexylcarbodiimide (DCC) and NHS in THF. To a 38.8 mM solution of LA (23.9 mg, 0.116 mmol) in 3.0 mL of THF at 0 °C, 1 equivalent of NHS (13.3 mg, 0.116 mmol) and 1 equivalent of DCC (23.9 mg, 0.116 mmol) were added. The reaction mixture was kept under nitrogen and stirred for 30 min at 0 °C, and then allowed to react for 48 h at room temperature. The reaction product was purified by means of centrifugation cycles, to remove dicyclohexylurea (DCU). In a first step, the DCU was removed and the supernatant solution was dried under vacuum. The pellet was then dissolved in acetone, and the solution centrifuged to remove residual DCU. This procedure was repeated for five cycles. Finally, the product was obtained as a yellow solid upon recrystallization from acetone/hexane (1/4 *v*/*v*). In total, 15.25 mg (43.4% yield) of the *N*-hydroxysuccinimidyl lipoate were obtained. The *N*-hydroxysuccinimidyl lipoate was then added to MIMO in dimethylformammide (DMF) solution, to give MIMO@LA. Next, 15.0 mg of MIMO (3.88 µmol, 1.29 mM) were allowed to react with 10 equivalents (11.7 mg, 38.8 µmol, 12.9 mM) of *N*-hydroxysuccinimidyl lipoate in DMF (3.0 mL), in the presence of 4 equivalents of diisopropylethylamine (DIEA). The mixture was allowed to react overnight. The reaction product was purified by RP-HPLC, and purity and the identity of the product was determined by RP-HPLC-ESI-MS (see Supplementary Materials). The chromatographic profile of the reaction product showed the presence of a main peak with a t_R_ = 25.6 min (Figure S4). ESI-MS mass analysis allowed identifying the product as the desired MIMO@LA (experimental mass 3709.2 Da, theoretical mass 3709.53 Da). The product was obtained with a 34.0% yield.

### 3.2. Synthesis and Characterization of MIMO@LA@AuNPs

Citrate-stabilized gold nanoparticles (AuNPs) were prepared by citrate reduction of HAuCl_4_ according to the procedure developed by Turkevich [70]. An aqueous solution of HAuCl_4_ (1 mM, 25.5 mL) was brought to boil, and then kept at reflux under stirring for 10 min. Once the gold solution was vigorously refluxing, an aqueous solution of sodium citrate (39 mM, 2.54 mL) was added quickly. Upon the addition of the sodium citrate, the color of the solution turned ruby-red, indicating the formation of the gold nanoparticles. The suspension was allowed to heat for additional 30 min with vigorous stirring. The suspension was then allowed to cool to room temperature. The AuNPs solution was stored at 4 °C in the dark for subsequent uses. 

Conjugation of MIMO@LA to AuNPs was obtained by substitution of the citrate ligands with lipoic acid, following the procedure reported by Onoda et al. [65]. The pH of the stock citrate-stabilized AuNPs solution was adjusted to 11 with the dropwise addition of 1 M NaOH. To 2.8 mL of this stock solution (AuNPs concentration ≅ 8 nM), a mixture of MIMO@LA (0.600 mg, 1.62 × 10^−4^ mmol, in 400 µL) and LA (0.204 mg, 9.93 × 10^−4^ mmol, in 13.6 µL of 15 mg/mL solution) in DMSO (LA:MIMO@LA ratio ≅ 6:1) was added. The solution was incubated at room temperature in the dark, and the proceeding of the reaction was followed by UV-Vis analysis, evaluating the shift in the SPR band upon MIMO@LA conjugation. After 3 h, the solution was centrifuged at 12,000 rpm for 25 min at 4 °C. To remove excess MIMO@LA and LA, the supernatant was decanted and the remaining pellet was re-dispersed in NaOH solution (pH 11). This procedure was repeated three times to obtain purified MIMO@LA@AuNPs. The pellet was then resuspended in NaOH solution (pH 11), and promptly used for subsequent experiments.

### 3.3. MIMO Radius of Gyration Calculation

The radius of gyration (R_G_) [76] for MIMO molecule was calculated on the model structure (see Figure S7), by using the following equation:

(2) $$R_{G}=\sqrt{\frac{1}{N}}\overset{N}{\underset{i=1}{\sum }}{\left({\vec{x}}_{i}-\vec{x}\right)}^{2}$$

where ${\vec{x}}_{i}$ is the position of the atom *i* in the model structure, *x* is the position of the centroid of the molecule, and *N* is the total number of non-hydrogen atoms. Calculation afforded a value of 9.03 Å (0.903 nm) for MIMO *R_G_* value. 

### 3.4. AuNPs Physico-Chemical Characterization

Transmission electron microscopy (TEM) images were obtained in the bright-field mode using a Philips EM 208S TEM (Philips, Eindhoven, The Netherlands), with an accelerating voltage of 100 kV. The samples were prepared by evaporating two drops (5 μL) of either AuNPs or MIMO@LA@AuNPs solutions onto carbon-coated copper grid (200 mesh) and allowing them to dry at room temperature overnight. To observe the protein shell by TEM, MIMO@LA@AuNPs conjugates were analyzed upon negative staining with 1% uranyl acetate. The histograms of the particle size distribution and the average particle diameter were calculated from the TEM images by using ImageJ software (National Institutes of Health, available free of charge at Web site rsb.info.nih.gov/ij/). At least 50 independent measurements were taken at different locations of the TEM images of the samples. The measurements were also confirmed by repeating the analysis on TEM images of independent samples.

Concentrations of the prepared AuNPs solution was estimated as reported in the literature [84]. By assuming that all the initial gold is incorporated into spherical particles with the density of the bulk gold (*ρ*_gold_ = 19 g/cm^3^) [85], AuNPs concentration can be obtained by dividing the initial 1 mM concentration of atomic gold by the following equation:

(3) $$\frac{4}{3}\pi r^{3}\;\frac{\rho_{gold}}{AW_{gold}}\;A$$

where *r* is the average radius of the particles from TEM data, A is Avogadro’s number, and *AW*_gold_ is the atomic weight of gold (197.97 g/mol). By considering *r* = 8.0 nm, as derived from TEM analysis, AuNPs concentration was assessed to be 8.06 nM.

The concentration of AuNPs in the stock solution was also determined directly from UV-vis spectra, by using the method reported by Fernig and coworkers [86]. As reported, the ratio of the absorbance at the SPR band (A_SPR_) to the absorbance at 450 nm (A_450_) can give an indication of the AuNPs diameter. For an A_SPR_/A_450_ ratio of 1.64, the diameter of AuNPs was calculated to be ≅16 nm, in good agreement with TEM data (Figure S6a). From the value of A_450_ and using the calculated extinction coefficient ε_450_ of 2.67 × 10^8^ M^−1^ cm^−1^, AuNPs concentration in the stock solution was estimated to be 7.43 nM, in good agreement with the previous calculation.

### 3.5. Voltammetric Analysis

All cyclic voltammetry experiments were performed with a Potentiostat/Galvanostat μAUTOLAB Type III (Ecochemie, Utrecht, The Netherlands) using a three-electrode cell for small volume samples (0.5–1 mL) purchased from BASi, under argon. Temperature controlled measurements were conducted using a thermo-cryostat R2 (Grant Instruments, Cambridge, UK). For all voltammetric analyses, argon gas was purged through the solution for at least 20 min to remove any dissolved oxygen before every experiment. An argon atmosphere was maintained over the solution during the measurements. A 3 mm-diameter GCE was used as working electrode, for the freely diffusing species. Measurements were carried out in 5.0 mM phosphate buffer, pH 7.0, with 100 mM NaClO_4_ as the supporting electrolyte. For electrochemical measurements of the immobilized enzyme, MIMO@LA was immobilized onto a 1.6 mm-diameter polycrystalline gold electrode through covalent binding via S-Au bond of the LA moiety. A clean gold electrode was immersed into a freshly prepared 9.0 mM solution of MIMO@LA in ethanol/water 75/25 (*v*/*v*) for 48 h at 4 °C. The electrode was rinsed with deionized water and then immersed into phosphate buffer solution (10.0 mM, pH 7.0) for electrochemical measurements.

Platinum wire and Ag/AgCl (3.0 M NaCl) electrodes were used as counter and reference electrode, respectively. A Vycor (PAR) set ensured the electric contact between the Ag/AgCl electrode and the working solution. Glassy carbon electrodes were polished mechanically with an abrasive alumina powder [87]. Gold electrodes were treated with HNO_3_ 3 M, by dropping 5 μL of the acid on the surface of the electrode. After 15 min, electrodes were rinsed thoroughly with deionized water, and then the same polishing procedure described above for GCE was applied. The degree of polishing of gold electrodes was evaluated by looking at the difference in the peak potential for the redox couple of Ferri/Ferrocyanide, which is expected to be close to 60 mV [88]. A 10 mM ferricyanide solution in ethanol/0.1 M NaClO_4_ 50/50 (*v*/*v*) was used to register a cyclic voltammogram at a scan rate of 0.05 V/s. The estimation of the surface coverage of the electrode was made according to literature methods [67]. According to this method, the peak current (*I_p_*) is related to the surface concentration of the electroactive species, *Γ*, by the following equation:

(4) $$I_{p}=\frac{n^{2}F^{2}\;A\Gamma v}{4RT}$$

where *n* represents the number of electrons involved in the reaction, *A* is the surface area (2.01 × 10^−2^ cm^2^) of the electrode, *Γ* (mol·cm^−2^) is the surface coverage, and the other symbols have their usual meanings. From the slope of the anodic peak currents versus the scan rate (Figure 2c), the calculated surface concentration was 20.4 pmol·cm^−2^ for *n* = 1.

### 3.6. Catalytic Assays

The catalytic activity of the MIMO@LA@AuNPs was assayed as for the freely diffusing enzyme. All the reactions were carried out in phosphate buffer (50 mM, pH 6.5), in the presence of 50% TFE (*v*/*v*). All the catalytic experiments were followed using a Varian Cary 50 spectrophotometer, by using ABTS [89] as substrate. The change in absorbance of the ABTS^+●^ cation radical was followed at 660 nm (*λ*_max_ = 660 nm, *ε*_660_ = 1.40 × 10^4^ M^−1^cm^−1^). Kinetic parameters were determined by varying H_2_O_2_ concentration using fixed concentrations of reducing substrate, and vice versa.

In the experiments performed at variable H_2_O_2_ concentration (in the range 1.0–100 mM), the ABTS concentration was kept constant at 5.0 mM. In the experiments performed at variable ABTS concentration (in the range 0.01–10.0 mM) the H_2_O_2_ concentration was 30 mM. For the freely diffusing experiments MIMO concentration was 2.0 × 10^−7^ M and reaction volume was 1.0 mL. For the MIMO conjugated to AuNPS, in a typical assay, 33.0 µL of the MIMO@LA@AuNPs solution were diluted to a final volume of 1.0 mL to afford a 3.2 × 10^−7^ M concentration of the enzyme. The kinetic parameters were determined using the two-substrate Michaelis–Menten kinetic model [90]. Data were analyzed using the following equation:

(5) $$v=\frac{{\left[E\right]}_{0}}{\frac{1}{k_{cat}}+\frac{K_{M_{A}}}{k_{cat}\left[A\right]}+\frac{K_{M_{B}}}{k_{cat}\left[B\right]}}$$

where *v* is the initial rate, [*E*]_0_ is the enzyme concentration, [*A*] is the H_2_O_2_ concentration, and [*B*] is the ABTS concentration.

## 4. Conclusions

In this work, we demonstrated that artificial peroxidases such mimochromes are promising candidates to be used for different applications as immobilized enzymes. Through the modification of the MIMO artificial enzyme with a specific linker such LA, a redox- and catalytically-active biomolecular component can be easily and rapidly grafted onto different surfaces such as gold electrodes or AuNPs. Upon immobilization onto gold electrode, MIMO@LA produced a hybrid interface allowing for direct electron transfer between the electrode and the redox-active heme-center. This behavior is very important for the future development of mimochrome-modified electrode surfaces useful in the construction of electrochemical biosensors. Further, conjugation of MIMO@LA to AuNPs afforded catalytically active MIMO@LA@AuNPs. Even though a decrease in MIMO catalytic efficiency was observed upon conjugation, these results represent a proof of concept in employing mimochrome artificial peroxidases as bioactive component of AuNP-based biomaterials. The use of artificial enzymes will expand the collection and the applicability of gold-based biomaterials, because the biomolecular component can be tailored to specific functions. In conclusion, this study is prodromal to the development of new gold-based biomaterials, which can find applications in catalysis, biosensors and immune-enzymatic diagnostic assays.

## Figures and Tables

**Table 1:** Kinetic parameters for H_2_O_2_ dependent oxidation of ABTS by MIMO@LA@AuNPs. The data for the free MIMO enzyme are also reported for comparison (*k*_cat_: turnover frequency; *K*_M_: Michaelis–Menten constant; MC6: Fe(III)-Mimochrome VI).

Compound	*k*_cat_ (s^−1^)	*K*_M_ ABTS (10^−5^ M)	*k*_cat_/*K*_M_ (mM^−1^·s^−1^)
MIMO@LA@AuNPs	1.7± 0.1	14.7 ± 0.1	11.5
MIMO	468 ± 27	12.5 ± 2	3743 ± 814
MC6^§^	371 ± 14	8.4 ± 0.2	4417 ± 197

^§^ from Ref. [50].

## Supplementary Materials

Supplementary Materials can be found at http://www.mdpi.com/1422-0067/19/10/2896/s1.
