# Supplementary material for: Artificial Heme Enzymes for the Construction of Gold-Based Biomaterials
Source: Int J Mol Sci. 2018 Sep 24;19(10):2896. doi: 10.3390/ijms19102896 (PMC6213134; doi:10.3390/ijms19102896)
Supplement: Supplementary file 1 [file ijms-19-02896-s001.zip › ijms-343491-SI.pdf]

SUPPLEMENTARY MATERIAL

Artificial heme enzymes for the construction of gold-based biomaterials

Gerardo Zambrano [1], Emmanuel Ruggiero [1,†], Anna Malafronte [1], Marco Chino [1], Ornella Maglio [1,2], Vincenzo Pavone [1], Flavia Nastri [1] and Angela Lombardi [1,*]

[1]Department of Chemical Sciences, University of Napoli "Federico II" Via Cintia, 80126 Napoli, Italy; gerardo.zambrano@unina.it (G.Z.); emmanuel.ruggiero@basf.com (E.M.); anna.malafronte@unina.it (A.M.); marco.chino@unina.it (M.C.); ornella.maglio@unina.it (O.M.); vincenzo.pavone@unina.it (V.P.); flavia.nastri@unina.it (F.N.); alombard@unina.it (A.L.)

[2]Istituto di Biostrutture e Bioimmagini, CNR, Via Mezzocannone 16, 80134 Napoli, Italy; ornella.maglio@unina.it

* Correspondence: alombard@unina.it, Tel.: +39-081-674418; flavia.nastri@unina.it, Tel.: +39-081-674419

† Current address: BASF SE, Dept. Material Physics, Carl-Bosch-Strasse 38, 67056 Ludwigshafen, Germany

Table of Contents

S1. Materials and instrumentation 3

S2. Experimental procedures 3

S2.1 Synthesis of MIMO 3

S2.2 Synthesis of MIMO@LA 6

S3. Figures S5 - S9 8

Figure S5 (Cyclic voltammogram of MIMO@LA) 8

Figure S6 (Particle size distribution histograms) 9

Figure S7 (Surface representation of MIMO@LA model) 10

Figure S8 (Visible spectrum of MIMO@LA@AuNPs) 11

Figure S9 (Peroxidase activity of freely diffusing MIMO) 12

S4. References 12

S1. Materials and instrumentation

All Fmoc (9-fluorenylmethoxycarbonyl) protected amino acids, NovaSyn TG Sieber resin, and coupling reagents (HOBt, 1-Hydroxybenzotriazole, HATU, 1-[Bis(dimethylamino)methylene]-1H-1,2,3-triazolo[4,5-b]pyridinium 3-oxid hexafluorophosphate, and PyBOP, (Benzotriazol-1-yloxy)tripyrrolidinophosphoniumhexafluorophosphate) were purchased from Novabiochem. All solvents, used in the synthesis and purification, were anhydrous and HPLC grade, respectively, and were supplied by Romil. Piperidine and scavengers (ethanedithiol, triisopropylsilane) were from Fluka. TFA (trifluoroacetic acid) and DIPEA (N,N-Diisopropylethylamine) were from Applied Biosystems. Pre-coated silica G-60 plates, F254, used for thin layer chromatography (TLC), were from Merck. Deuteroporphyrin IX was from Porphyrin Products. Iron (II) acetate was purchased from Sigma Aldrich, as well as and DCC (*N*,*N'*-Dicyclohexylcarbodiimide).

Protected peptides were obtained by the use of ABI 433 automatic peptide synthesizer (Applied Biosystems, Foster City, CA, USA). HPLC and LC-MS analysis were performed with a Shimadzu LC-10ADvp equipped with an SPDM10Avp diode-array detector. Solvent mixtures are indicated in the respective sections. ESI-MS spectra were recorded on a Shimadzu LC-MS-2010EV system with ESI interface, Q-array-octapole-quadrupole mass analyzer, and Shimadzu LC-MS solution Workstation software for data processing. The optimized MS parameters were selected as followed: CDL (curved desolvation line) temperature 250 °C; the block temperature 250 °C; the probe temperature 250 °C; detector gain 1.6kV; probe voltage +4.5kV; CDL voltage -15V. Nitrogen served as nebulizer gas (flow rate: 1.5 L/min).

Flash Chromatography was performed using a Biotage Isolera flash purification system, equipped with a diode-array detector. UV-vis analysis was performed on Cary Varian 50 Probe UV Spectrophotometer. All the data were analyzed by using the Origin Pro 8 and the Kaleidagraph software.

S2. Experimental procedures

S2.1 Synthesis of MIMO (Fe(III)-Mimochrome VI Ser6Gly)

Fe(III)-Mimochrome VI Ser6Gly (herein refereed as MIMO, Figure S1) was synthesized combining solution and solid-phase peptide methods, as reported for previous Mimochrome analogues.[1,2]

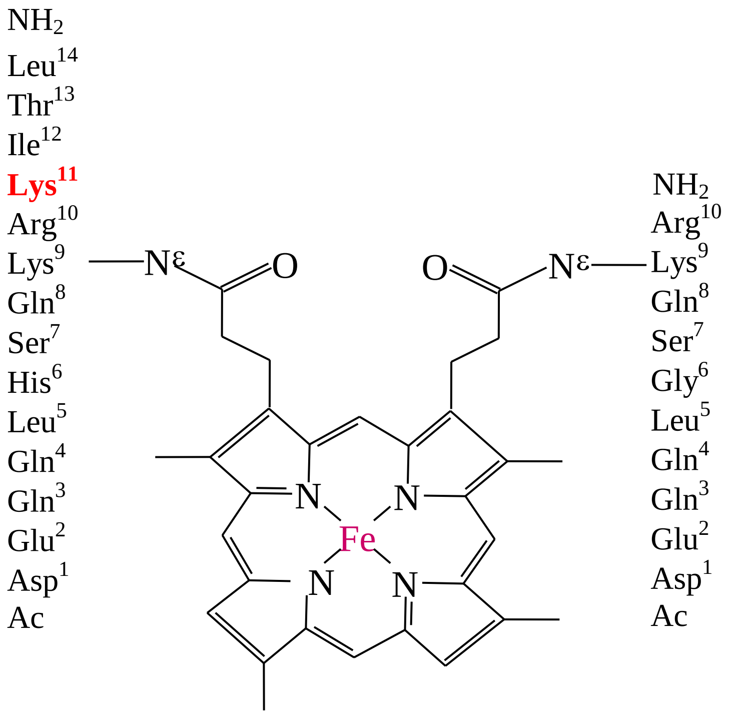

Figure S1. Schematic representation depicting MIMO chemical structure. The Lys residue conjugated to LA is highlighted in red.

The two peptides, the tetradecapetide (TD) and the decapeptide (D) were synthesized by the solid-phase method using the 9-fluorenylmethoxycarbonyl (Fmoc) protection strategy, and they were coupled to deuteroporphyrin in solution to afford MIMO free base. Subsequently, iron ion was inserted, according to the acetate method procedure, slightly modified by us.[3,4] Iron (II) acetate (10 eq) was added to a solution of pure MIMO free base (2.0 x 10^{-4} M), in 2/3 TFE/AcOH (v/v), and the reaction mixture was kept at 50 °C for 2 h, refluxing under nitrogen. The reaction was monitored by analytical HPLC, using a Vydac C18 column, with an elution gradient of acetonitrile in 0.1% aqueous TFA, 5% to 80% over 35 min, at 1 mL/min flow rate. Once the reaction was completed, the solvent was removed under vacuum, and the product was purified to homogeneity by preparative RP-HPLC, following the procedure described above. After lyophilization, pure products were obtained as the TFA salt (yield 48%). LCMS analysis on an aliquot of the complex, obtained with high purity (>95%), confirmed the expected molecular weight (Figure. S2).

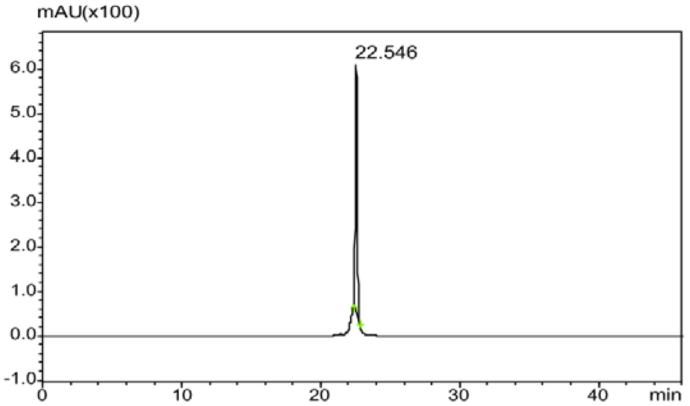

(a)

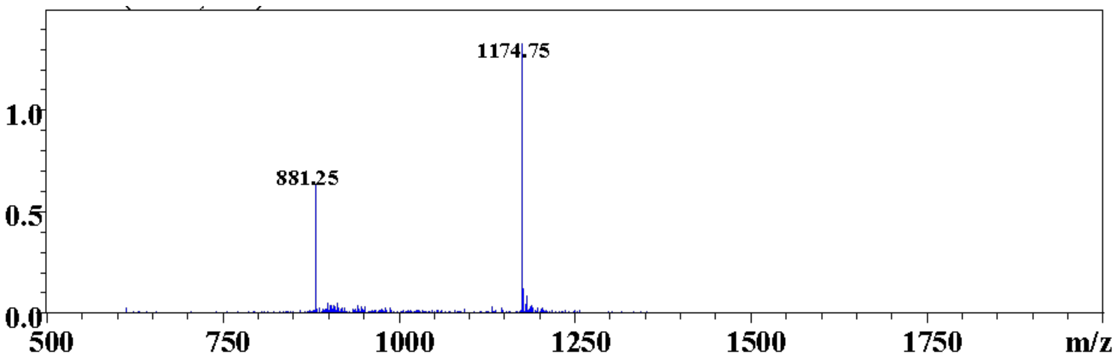

(b)

Figure S2. (a) LC-MS chromatogram of pure MIMO. (b) ESI-MS m/z spectrum of the peak at t_R=22.5 min.

The product at t_R =22.5 min was analyzed by ESI-MS spectrometry. Mass spectra confirmed the formation of the desired product (experimental *m/z* $[M+3H]^{3+}$ 1174.75; $[M+4H]^{4+}$ 881.25 were consistent with the theoretical mass 3521.2 Da).

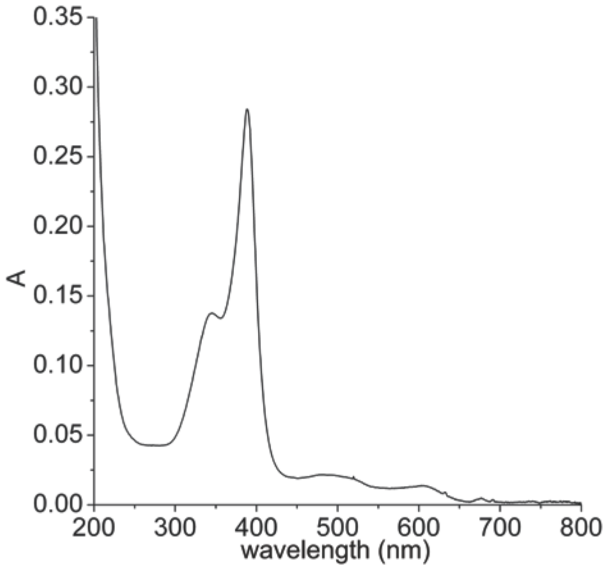

Figure S3. UV-Vis spectrum of MIMO (3.7×10^{-6} M) in 50 mM phosphate buffer pH 6.5 with 50% TFE (v/v); cell path length 1 cm.

The spectrum in Figure S3 is indicative of a His-H_2O axial coordination.

S2.2 Synthesis of MIMO@LA

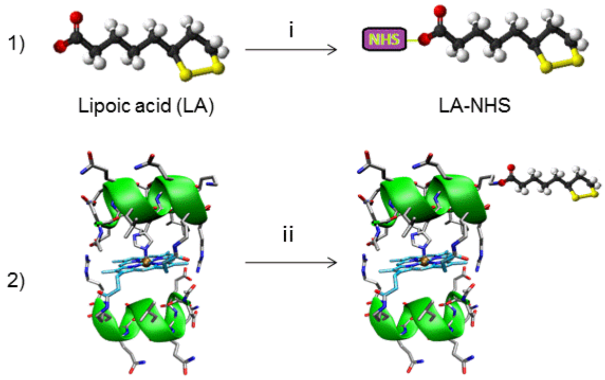

Scheme S1. Reaction Scheme for the synthesis of MIMO@LA. Reagents and conditions: (i) DCC, NHS, THF, 0°C; (ii) DMF, DIEA, LA-NHS.

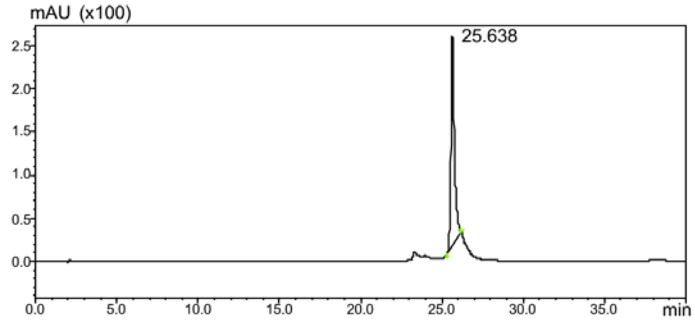

(a)

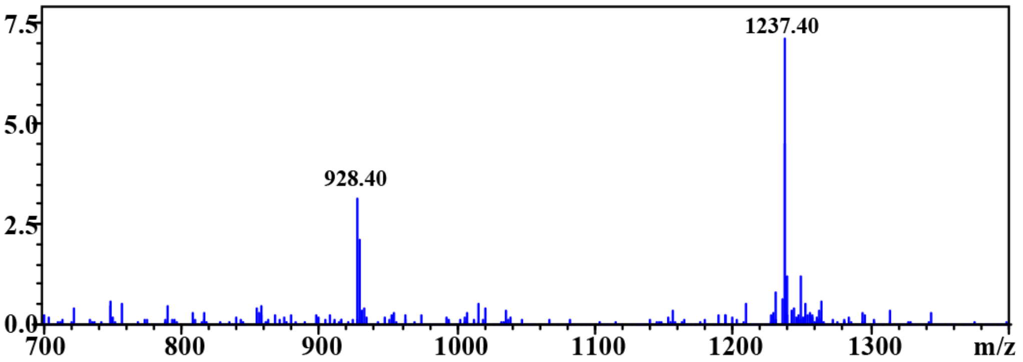

(b)

Figure S4. (a) LC-MS chromatogram of pure MIMO@LA. (b) ESI-MS m/z spectrum of the peak at t_R=25.6 min.

The product at t_R =25.6 min was analyzed by ESI-MS spectrometry. Mass spectra confirmed the formation of the desired product (experimental *m/z* $[M+3H]^{3+}$ 1237.4; $[M+4H]^{4+}$ 928.40 were consistent with the theoretical mass 3709.53 Da).

S3. Figures S5 - S9

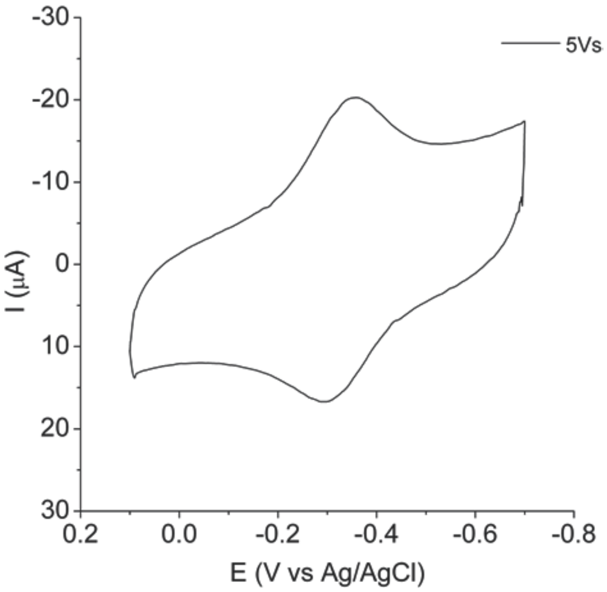

Figure S5. Cyclic voltammogram of MIMO@LA freely diffusing in 10.0 mM phosphate buffer solution, pH 7.0, at *v* 5.0 V/s. Measurements are reported vs Ag/AgCl electrode.

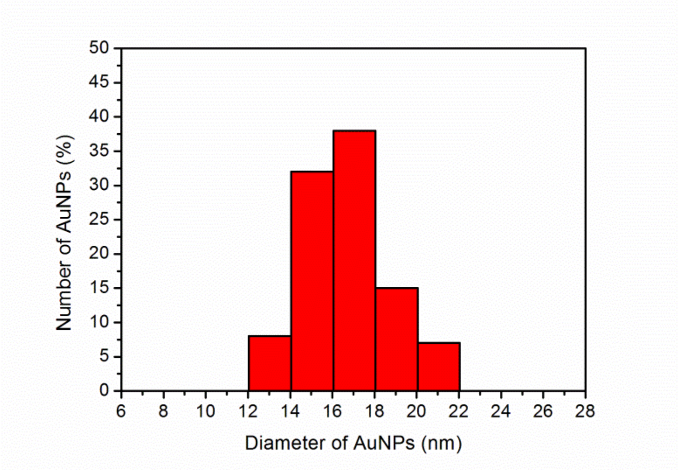

(a)

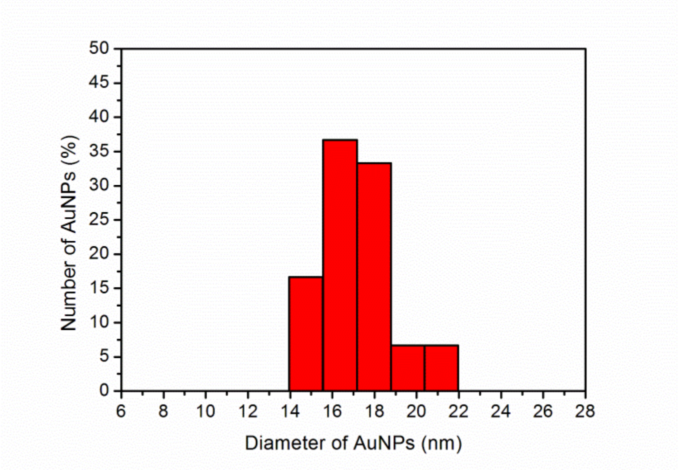

(b)

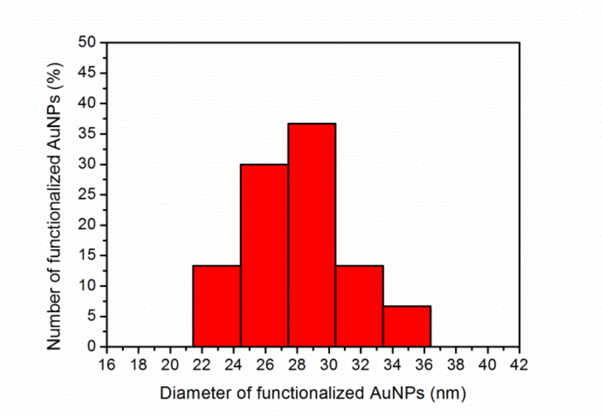

(c)

Figure S6. Particle size distribution histograms of AuNPs as derived from TEM images. a) Citrate-capped AuNPs. b) MIMO@LA@AuNPs by measuring the AuNPs gold core. c) MIMO@LA@AuNPs by measuring the AuNPs gold core *plus* the protein shell.

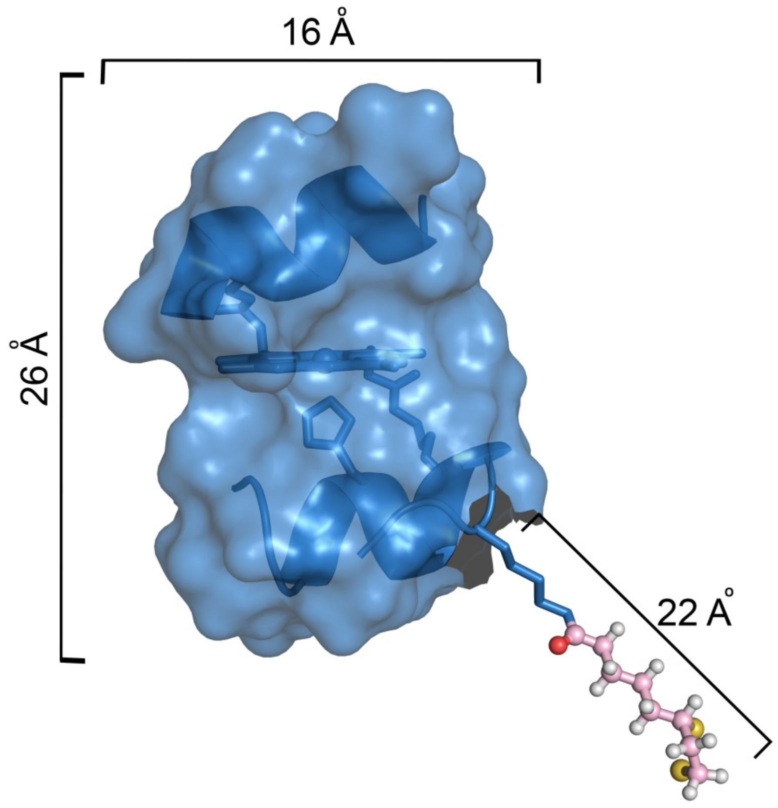

Figure S7. Surface representation of MIMO@LA model, with indications of height and base diameter of the cylindroid structure. The length of the linker made up of the side chain of Lys and LA (considering both chains in an all-*trans* extended conformation) is also indicated. The model structure was generated with PyMol.[5]

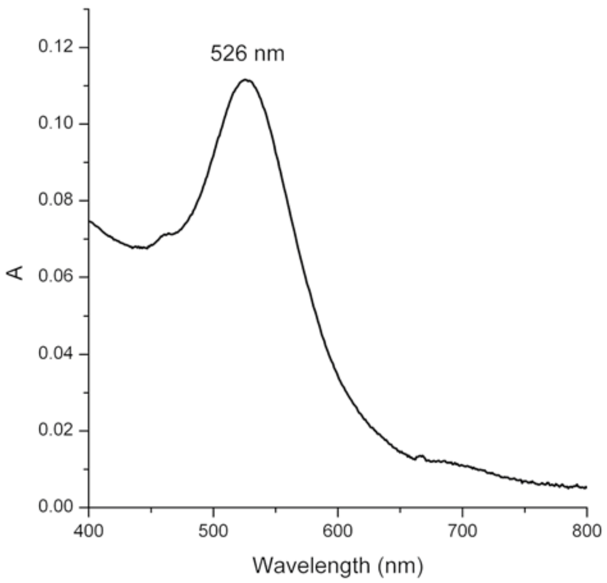

Figure S8. Visible spectrum of MIMO@LA@AuNPs suspension in 50 mM phosphate buffer pH 6.5 50% TFE (v/v).

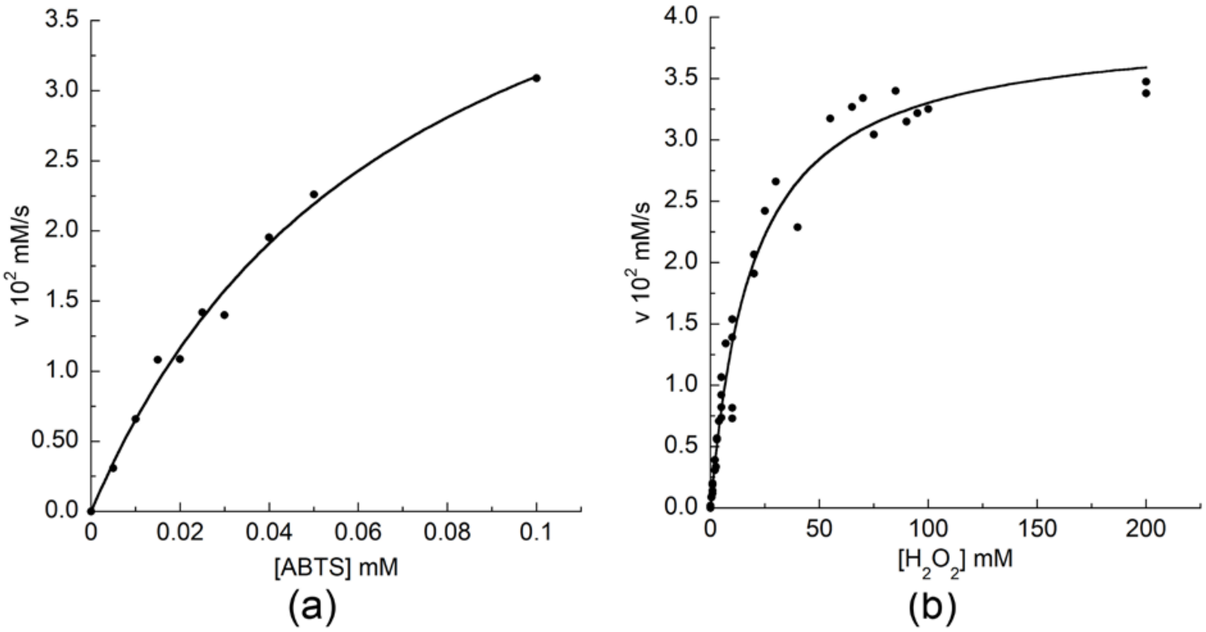

Figure S9. Peroxidase activity of freely diffusing MIMO. (**a**) Initial rate dependence towards ABTS concentration. (**b**) Initial rate dependence towards H_2O_2 concentration. Reaction conditions were: 50 mM phosphate buffer pH 6.5 50% TFE (v/v); MIMO concentration 2.0 x 10^{-7} M. For the experiments performed at variable ABTS (a) the H_2O_2 concentration was fixed at 50 mM For the experiments performed at variable H_2O_2 (b) the ABTS concentration was fixed at 5.0 mM.

S4. References

[1] Nastri, F., Lista, L., Ringhieri, P., Vitale, R., Faiella, M., Andreozzi, C., Travascio, P., Maglio, O., Lombardi, A., and Pavone, V. (2011) *Chem. Eur. J. 17*, 4444-4453.

[2] Vitale, R., Lista, L., Cerrone, C., Caserta, G., Chino, M., Maglio, O., Nastri, F., Pavone, V., and Lombardi, A. (2015) *Org. Biomol. Chem. 13*, 4859-4868.

[3] Buchler, J.W., in *The Porphyrins*, NY, USA, Academic Press., 1979, vol. I, pp. 389–483.

[4] F. Nastri, A. Lombardi, G. Morelli, O. Maglio, G. D’Auria, C. Pedone, and V. Pavone, *Chem. Eur. J.*, 1997, **3**, 340–349.

[5] PyMOL Molecular Graphics System, version 1.8, Schrödinger, LLC.
